# Effect of Cooling Rate at the Eutectoid Transformation Temperature on the Corrosion Resistance of Zn-4Al Alloy

**DOI:** 10.3390/ma13071703

**Published:** 2020-04-05

**Authors:** Marzena M. Lachowicz, Robert Jasionowski

**Affiliations:** 1 Faculty of Mechanical Engineering, Wroclaw University of Technology and Science, 50-370 Wrocław, Poland; 2 Faculty of Marine Engineering, Maritime University of Szczecin, 70-500 Szczecin, Poland; r.jasionowski@am.szczecin.pl

**Keywords:** zinc alloys, Zamak, Zn-Al alloys, heat treatment, corrosion resistance, microstructure

## Abstract

The main purpose of this work was to experimentally determine the effect of the cooling rate during the eutectoid transformation on the corrosion resistance of a hypoeutectic Zn-4Al cast alloy in 5% NaCl solution. This was considered in relation to the alloy microstructure. For this purpose, metallographic and electrochemical studies were performed. It was found that the faster cooling promoted the formation of finer (α + η) eutectoid structures, which translated into a higher hardness and lower corrosion current density. In the initial stage of corrosion processes the eutectoid structure in the eutectic areas were attacked. At the further stages of corrosion development, the phase η was dissolved, and the α phase appears to be protected by the formation of corrosion products.

## 1. Introduction

Zinc-based alloys have good tribological properties, relatively high mechanical strength and hardness values, and show good castability due to their low melting points. These features make them good candidates for use in automotive and electronics applications, and they have also been used in the production of small components and plain bearings. Studies have shown that these alloys have superior wear resistance to common copper-bearing alloys. Adhesion and smearing are the main wear mechanisms of zinc-based alloys, while abrasive wear is the predominant wear mode in bronzes [1,2,3,4,5]. In the last decade, zinc has been extensively studied as a potential biocompatible and biodegradable metal for medical applications [4,6,7,8,9,10].

Zinc has one of the lowest electrode potentials, and machines made from it are highly susceptible to electrochemical corrosion due to the formation of a galvanic cell. The presence of extensive corrosion may also affect other co-working components made of different materials. The resulting corrosion products affect the pH of the surrounding environment, which, in turn, may accelerate the degradation of lubricants. On the other hand, a low electrochemical potential gives zinc and its alloys broad application prospects as cathodic protection coatings. Thus, Zn-Al alloys may be used to replace traditional zinc galvanic coatings [11,12,13,14,15,16,17,18] The commercial Galfan alloy has found broad applications in this area [18] but it exhibits several serious drawbacks, including a low creep resistance, low shape stability associated with aging, insufficient corrosion resistance in acidic and alkaline environments, and a low cavitation erosion resistance [5,19,20,21]

**Figure 1:**
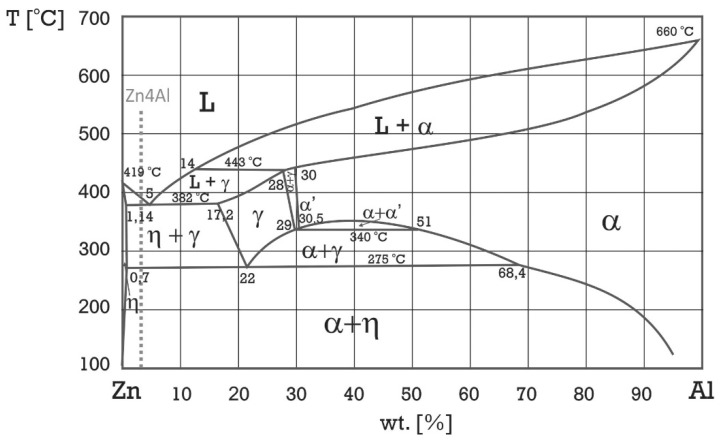
Zn-Al phase diagram adapted from [5].

The main alloying elements in Zn-based alloys are aluminum, magnesium, and copper. Cast Zn-Al alloys are commercially available under the Zamak tradename, the most popular of which is the Zamak 3 alloy which has a nominal composition of 4% Al. This aluminum content classifies this alloy as hypoeutectic (Figure 1) whose microstructure is composed of a η-Zn(Al) dendrite solid solution and (α+η) eutectic phases, in which the α solid solution is Al(Zn).

Chloride ions are one of the primary catalysts responsible for the corrosion of zinc and its alloys. Components made from these alloys are exposed to chloride ions in both seawater and also in seaside environments. Micron-sized salt aerosol particles can be deposited on elements located as far as 10 km from the shoreline. The threat in engineering practice may be intergranular corrosion by chloride ions [10,22,23], which was the main reason that a solution rich in these ions was used as a corrosive environment.

Analyzing the corrosion mechanisms of Zn-based alloys is challenging due to their complex microstructures. However, previous studies have only focused on the influence of factors affected by the crystallization conditions (from the temperature of the liquid phase). This translates into an effect on the size and branching of dendrites, as well as eutectic dispersion. Thus, practical considerations are important during casting. The grain size and microstructure morphology (affected by the crystallization conditions) [5,16,17,19,24] and cooling rate have been shown to affect the corrosion resistance of zinc alloys [12,24,25,26,27]. Finer dendrites were shown to improve the corrosion resistance of hypoeutectic alloys, whereas a coarse microstructure was more preferable for hypereutectic alloys [5,17]. The aim of this work was to determine the effect of the cooling rate during the eutectoid transformation on the corrosion resistance of a hypoeutectic Zn-4Al alloy. These changes apply to the crystallized alloy and are relevant to determining a heat treatment process. As a part of this research, samples were heat-treated at temperatures higher and lower than the eutectoid transformation, and electrochemical studies were combined with metallographic studies to confirm the effect of heat treatment on the alloy’s microstructure. The surface condition of the alloy was assessed after electrochemical tests to determine the role of microstructure during corrosion.

## 2. Materials and Methods

The investigated material was a Zn-4Al alloy that was fabricated by melting and casting pure elements (99.995% Zn and 99.7% Al) in a PIT10 induction furnace. The obtained material was subjected to heat treatment by annealing for 1 h at 250 °C and 300 °C, followed by cooling. The samples were quenched in water and cooled in air or in a furnace. The scheme of the research design is shown in Table 1.

Hardness measurements were performed using the Vickers method. Microscopic examinations were carried out using a stereoscopic microscope (Leica M205 C, Leica Microsystems, Wetzlar, Germany), a light microscope (Nikon Eclipse MA 200, Nikon Instruments Inc., Tokyo, Japan), and a scanning electron microscope (SEM) (Phenom World ProX, Thermo Fisher Scientific, Waltham, Massachusetts, USA). Light microscopy was used to examine metallographic sections to identify microstructural features after Nital etching (3% nitric acid in ethanol) and 5% NaCl solution. Stereoscopic and SEM microscopes were used after electrochemical measurements to illustrate the corrosion progress.

Polarization tests were performed using a three-electrode cell with a potentiostat (ATLAS 0531 ELEKTROCHEMICAL UNIT & IMPEDANCE ANALYSER, Atlas-Sollich, Gdansk, Poland). The auxiliary electrode was made of austenitic stainless steel, while a saturated Ag/AgCl electrode was used as the reference electrode. Just before the experiments, samples were subjected to mechanical grinding with 800 SiC emery papers. The surface area of the working electrode (the sample) was 0.785 cm^2^. Before experiments, each sample was immersed for 20 min in 250 mL of a 5% NaCl solution at room temperature. After that, the open circuit potential (E_OCP_) was measured. Polarization tests were conducted in the same solution by stepping the potential in the anodic direction using a scanning rate of 1 mV/s from −250 mV relative to the open-circuit potential. The pH of the applied solution was 7.5. Four anodic and cathodic polarization curves were recorded for the as-delivered material. The initial potential value was 200 mV lower than the E_OCP_ value. The polarization of each tested sample was terminated at different potential values. Thus, the potentiodynamic curves were stopped at potentials of +150, +225, +300, and +450 mV vs. E_corr_. Polarization curves were also obtained for samples heat-treated at 300 °C. Three curves per series were determined for the heat-treated alloy. The polarization curves were plotted using an automatic data acquisition system, and the corrosion potential (E_corr_) and corrosion current density (I_corr_) were estimated by Tafel plot extrapolation.

## 3. Results

### 3.1. Hardness Measurements

**Figure 2:**
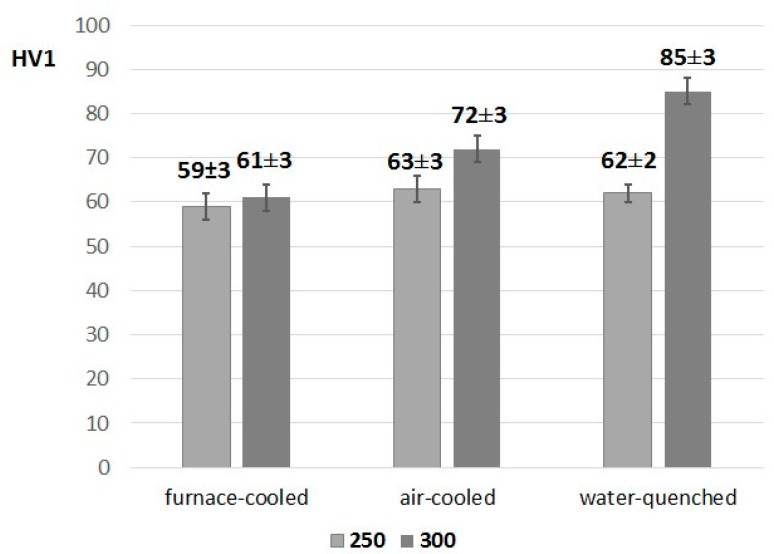
Hardness values obtained for heat-treated Zn-4Al alloy at 250 and 300 °C using various cooling rates.

The hardness measurement results and their standard deviations in Figure 2 show that the hardness of the as-delivered material was 58 ± 2 HV1. Changing the cooling rate affected the hardness of samples heat-treated at 300 °C. The hardness increased by more than 20 HV1 for the water-quenched sample compared with the material that was furnace-cooled from the same temperature (300 °C). This is due to the eutectoid transformation which occurred at 275 °C. Faster cooling promoted the formation of finer (α + η) eutectoid structures from the γ phase, while slower cooling allowed the alloy to form a coarser eutectoid structure, which translated into a lower hardness. The increased hardness due to the increased cooling rate realized from the beginning crystallization temperature and microstructure refinement has been observed by other Authors [27].

To provide a comparison, heat treatment was also carried out at a temperature lower than the eutectoid transformation, i.e., 250 °C, and various cooling rates were also used. The cooling rate had no effect on the material hardness at this temperature, which indicates that the formed microstructure was stable. The slight differences in the hardness values were within the standard deviation.

### 3.2. Microstructural Examination

**Figure 3:**
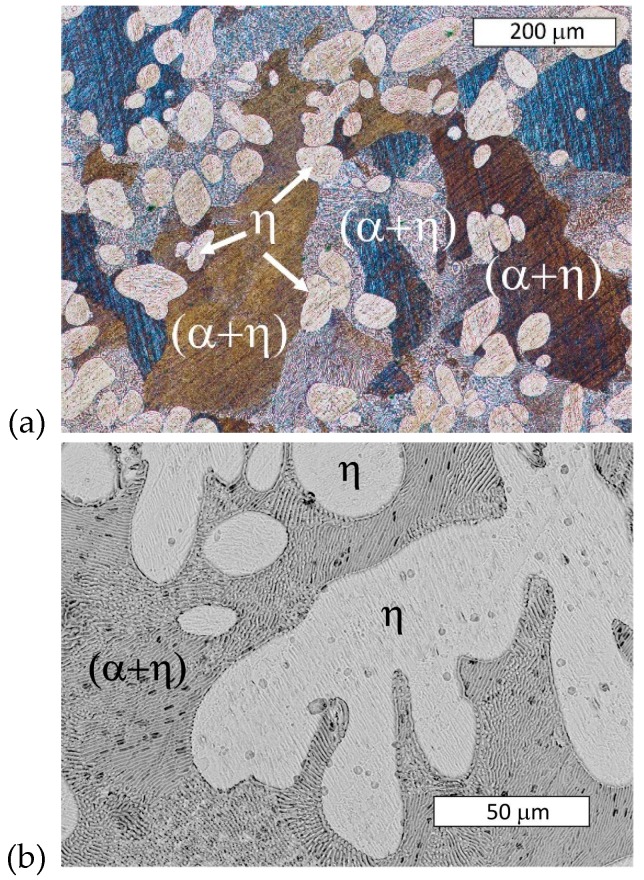
Microstructure of examined Zn-4Al alloy (as-delivered). Visible dendrites of η phase and a eutectic lamellar morphology (α + η). Etched with 10% NaCl solution. (**a**) Light Microscopy, (**b**) SEM.

The microstructure of the material in delivered state was typical of hypoeutectic Zn-Al alloys (Figure 3). Dendrites of the Zn-base solid solution (η) and an (α + η) eutectic lamellar structure were visible. The microstructure contained the product of eutectoid decomposition because the γ phase was transformed into (α + η) phase at 275 °C, as shown in Figure 1. On the other hand, rod-like eutectic features that may have been formed due to rapid quenching were not observed [11].

**Figure 4:**
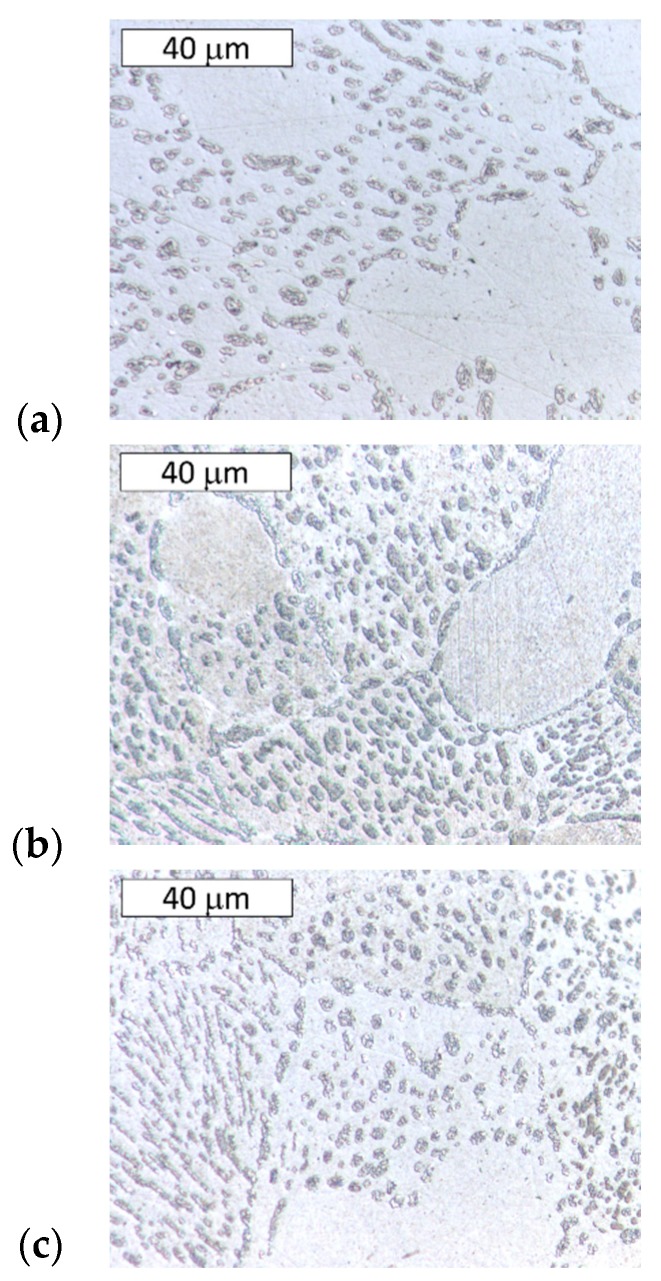
Microstructure of Zn-4Al alloy after heat treatment at 300 °C: (**a**) furnace-cooled, (**b**) air-cooled, (**c**) water-quenched. The visible morphology of the eutectic structure was not affected by the eutectoid transformation. Etched with Nital. Light Microscopy.

**Figure 5:**
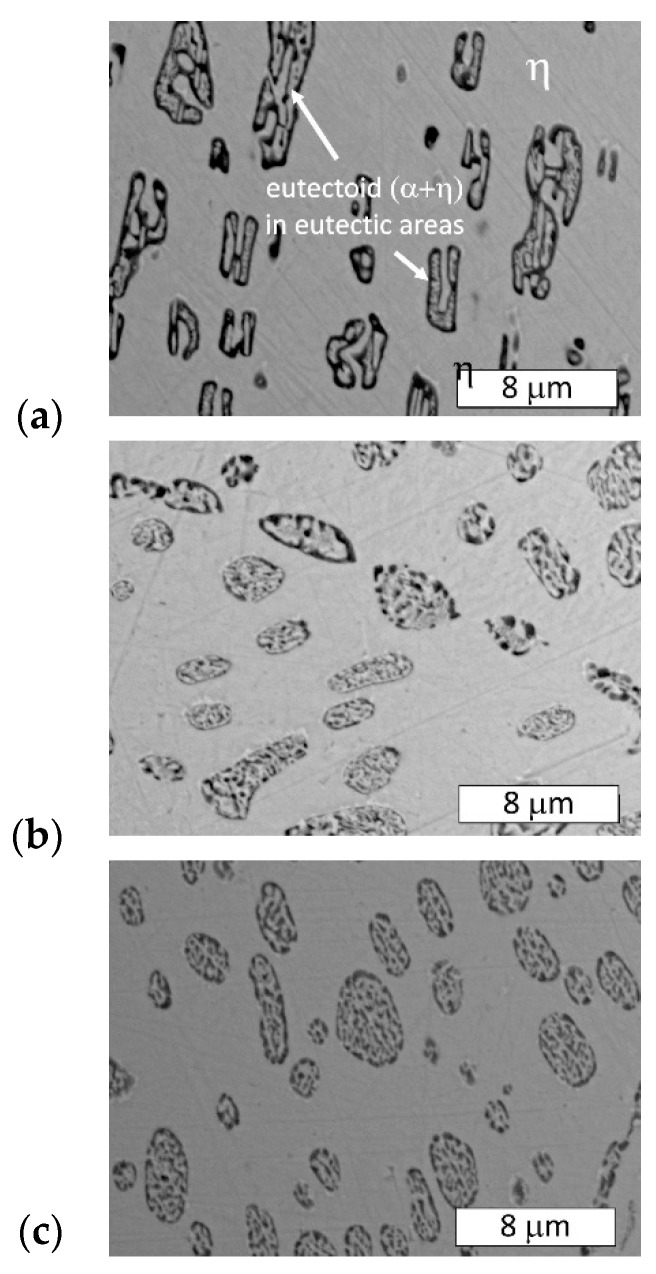
Microstructure of the Zn-4Al alloy after heat treatment at 300 °C: (**a**) furnace-cooled, (**b**) air-cooled, (**c**) water-quenched. A finer (α + η) eutectoid phase was formed from the γ phase. Etched with Nital. SEM.

After heat treatment at 300 °C, the effect of the cooling rate on the phase distribution in the eutectoid structure was examined (Figure 4 and Figure 5). During the applied heat treatment, only the morphology of the microconstituents inside the lamellar structure was affected by eutectoid decomposition. Some divorced eutectic structure was also observed along the grain boundaries. Despite the eutectoid decomposition, the morphology of the interdendritic lamellar eutectic structure was not affected because it was not subjected to any solid-state transformation (Figure 4).

### 3.3. Electrochemical Examinations

**Figure 6:**
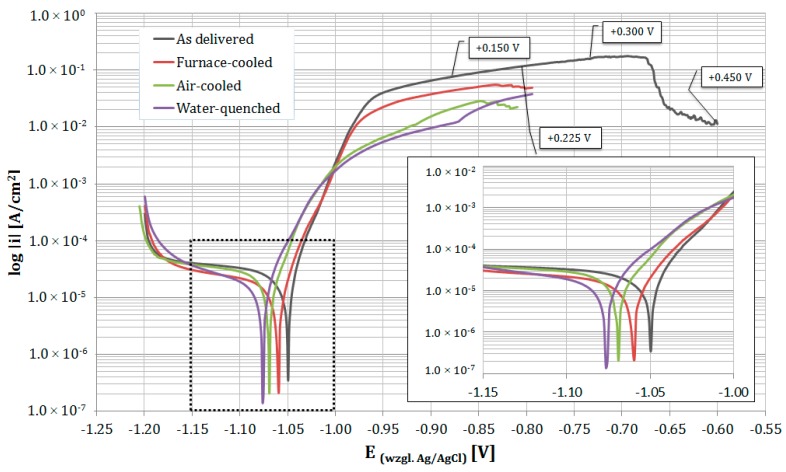
Example potentiodynamic polarization curves of the as-delivered Zn-4Al alloy and heat-treated at 300 °C in 5% NaCl solution. In the curve of the sample polarized to the highest potential value, the potential values (relative to E_corr_) were marked where polarization was stopped.

Figure 6 shows a comparison between the polarization curves of investigated samples. The corrosion current density and corrosion potential were estimated from the polarization curves using the Tafel extrapolation method (Table 2). The corrosion test results of the as-delivered material are presented as the average of four measurements. For the heat-treated samples measurements are shown as the average of three measurements. As expected, the Zn-4Al alloy had a negative corrosion potential, and the four curves obtained for the as delivered material were similar. This value was consistent with the results of other Authors [6,16]. A strong increase in the current density during the initial stage of the anodic curve was found, which indicates highly intense electrochemical processes.

The microstructure of the investigated material was composed of two phases—an α aluminum-rich solid solution and a η zinc-rich solid solution. The resultant electrochemical potential was closely related to the phase heterogeneity of the zinc alloy, i.e., to the corrosion potential of each phase. The phases with various electrode potentials became anodic and cathodic during contact between the alloy and the electrolyte [28]. Al has a nobler electrochemical behavior than Zn [17,29], and similar behavior should be attributed to Al-base and Zn-base solid solutions. It was previously shown that the anodic nature of the η phase depends on the pH of the corrosive agent [16,24,30]. In slightly acidic or neutral environments, the α phase is nobler than the η phase, so it may act as a cathode. Conversely, in alkaline environments, the α phase may play the role of the anode [24,30]. Shihirova at al. [31] indicated that the electrochemical behavior of phases may be associated with local pH changing and their thermodynamic stability in this corrosive environment. In this study, experiments were carried out at a slightly alkaline pH of 7.5. However, the anodic processes lead to a local reduction pH due to the H^+^ produced from the hydrolysis of Al^3+^ [32].

The test results show that the corrosion current density and corrosion potential change as the microstructural morphology changes. The other morphologies were obtained due to different cooling rates during the eutectoid reaction. A very important factor in galvanic corrosion is the ratio of the anodic to cathodic area. If the surface of the cathode is larger than the anode, then more oxygen reduction or another cathodic reaction can occur, which increases the galvanic current. However, in this case, it remained at the same level, but the distance between the anode and cathode changed. 

In this case, we had a corrosion microcell, in which the anodes and cathodes were separated by just a few microns. Previous electrochemical research determined that finer structures show a lower I_corr_ compared with a coarse structure. E_corr_ remained rather constant, although it showed a slight decrease. It can be observed that the furnace-cooled structure was related to a corrosion current density and a corrosion potential of 7.01 µA/cm^2^ and −1.06 V (vs. Ag/AgCl), respectively, compared with 4.74 µA/cm^2^ and −1.07 V (vs. Ag/AgCl), respectively, for the water-cooled structure. Increasing the dispersion of cathode inclusions usually increases the cathode activity. However, if anode passivation occurs or a surface film of corrosion products forms, its activity can be decreased, and the anode process will be inhibited. On the other hand, the short phase distances typical of eutectoid structures may have protected the anode phase. This effect may be clearer due to the finer eutectoid structure.

The E_corr_ value was more electronegative than E_OCP_. The differences between the E_OCP_ and E_corr_ values were due to the diffusive nature of the cathode potential curve, which has been previously observed during anodic polarization [29].

### 3.4. SEM Surface Evaluation after Corrosion Tests

The surfaces of the as-delivered material after electrochemical study were examined using SEM. Samples whose anodic polarization was terminated at different potential values were examined in order to illustrate the corrosion progress in chloride-containing media. The results were discussed in relation to the structural features of the alloys.

**Figure 7:**
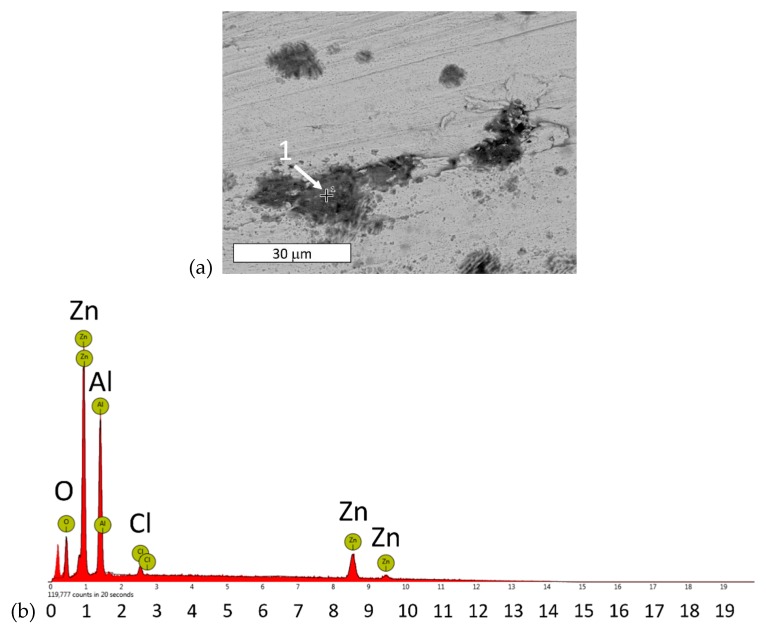
(**a**) SEM image of the surface of a sample after polarization up to a potential of +150 mV vs. E_corr_. Corrosion initiation areas are visible. The corrosion products are rich in aluminum and chlorine (marked with point 1 and summarized in Table 3; (**b**) characteristic X-ray emission spectrum obtained from point 1 in Figure 7a.

Corrosion began locally with the formation of aluminum-rich corrosion products (Figure 7, Table 3). The microscopic observations of the sample tested after reaching a potential of +150 mV versus E_corr_, did not permit the determination of which structural features underwent corrosion at this stage of development. However, the high aluminum content in the corrosion products on the surface suggested that degradation mainly involved eutectic areas. The formation of aluminum-rich corrosion products first may be unfavorable from the point of view using the alloy as a biomaterial.

Previous works have reported the preferential oxidation of Al-rich areas [29,33]. Other authors have shown that the α phase was protected at the initial stages of corrosion due to the formation of a corrosion product surface film that contained various aluminum-rich phases [24,34,35,36]. The presence of chlorine indicates that chlorides play an active role in the formation of corrosion products (Table 3). The simultaneous presence of Zn, Al, and Cl in the EDX spectra may be attributed to the formation of Zn_2_Al(OH)_6_Cl·2H_2_O, which has been reported to form during the early stage of corrosion [18]. Other Authors have observed an Al_2_(OH)_5_Cl·2H_2_O phase [35,36]. In this case, zinc may be associated with the base material. It is believed that, regardless of the chemical composition, these phases provide excellent protection against further corrosion. 

Based on the electrochemical tests and the above literature data, it can be hypothesized that the finer Al-base phase in the eutectoid structure may result in the formation of a more compact corrosion product film that increases the temporary corrosion protection. The formation of a corrosion product film on the α phase can help reduce the corrosion current density as the distance between eutectoid components decreases. Consequently, the finer distribution of the two phases that formed during eutectoid decomposition in the eutectic mixture tended to decrease their corrosion rate.

**Figure 8:**
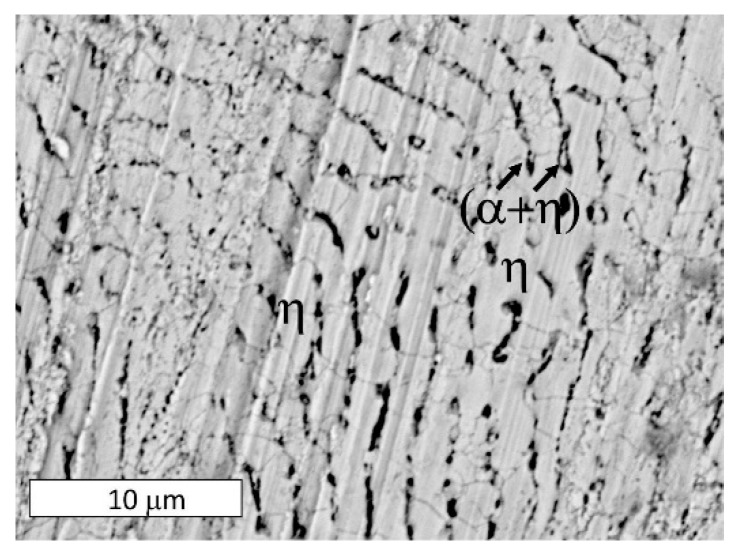
SEM image of the surface of a sample after polarization up to a potential of +225 mV vs. E_corr_. Selective dissolution of the eutectoid (α + η) in eutectic areas of the Zn-4Al alloy is visible.

**Figure 9:**
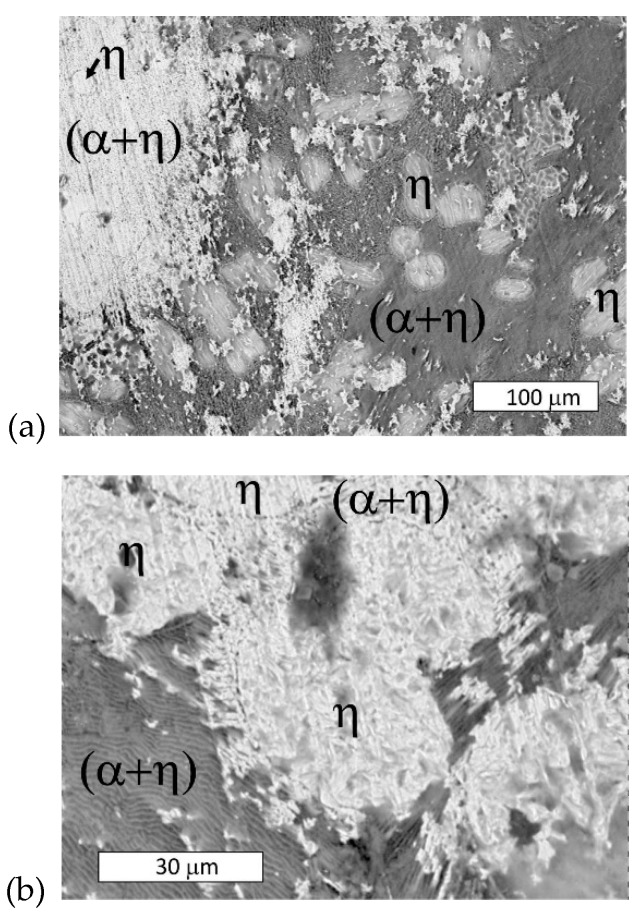
(**a**) SEM image of the surface of a sample after polarization up to a potential of +225 mV vs. E_corr_. Selective dissolution of the Zn-4Al alloy is visible. The dark areas represent areas in which the eutectic structure has dissolved. (**b**) Magnified image.

As corrosion progressed and the potential increased to +225 mV vs. E_corr,_ the alloy selectively dissolved. At this stage, due to the formation of an electrochemical cell between the α and η phases, the eutectoid (α + η) became susceptible to corrosion (Figure 8). Thus, the anode phase was present only in eutectoid areas, which suggested the α phase. When immersed in the corrosive solution, the hypoeutectic Zn-4Al alloy displayed Al-rich regions (the phase of the eutectic structure) which acted as anodic barriers that protected the η phase. Corrosion gradually occurred throughout the entire eutectic area (Figure 9), which was also reflected by a macroscopically visible color change over the sample surface where eutectics formed. The selective dissolution of eutectic areas has also been documented in other works [18,24,37]. Despite this, the local dissolution of η phase dendrites was also observed at higher magnifications (Figure 9a).

**Figure 10:**
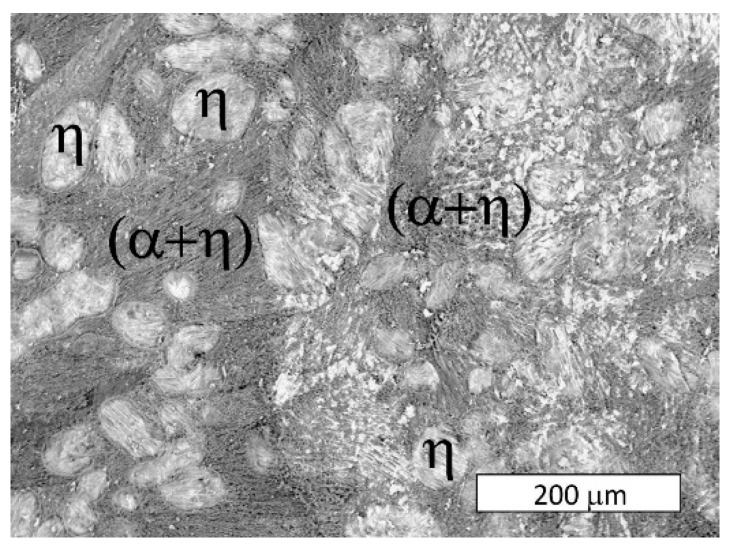
SEM image of the surface of a sample after polarization up to a potential of +300 mV vs. E_corr_. Corrosion initiation locations of the Zn-4Al alloy are visible. The dark areas represent areas of the eutectic alloy affected by corrosion.

**Figure 11:**
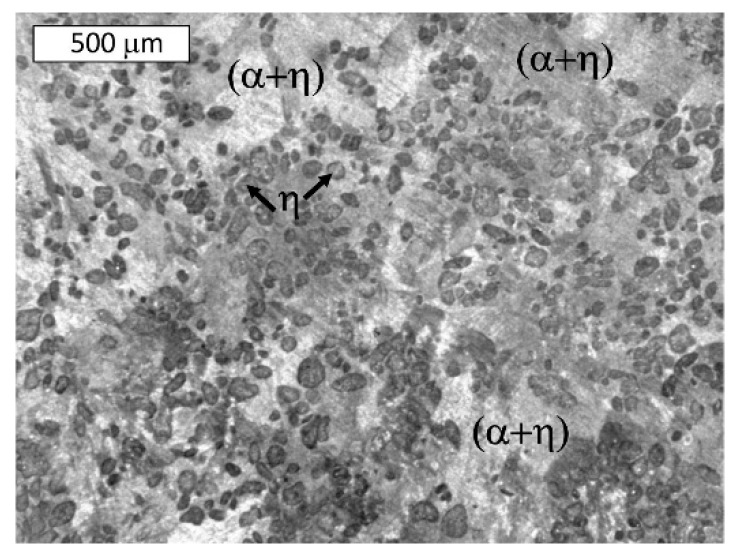
Stereoscopic image of a sample after polarization up to a potential of +300 mV vs. E_corr_. Selective dissolution of the dendritic η phase at the macroscopic scale.

**Figure 12:**
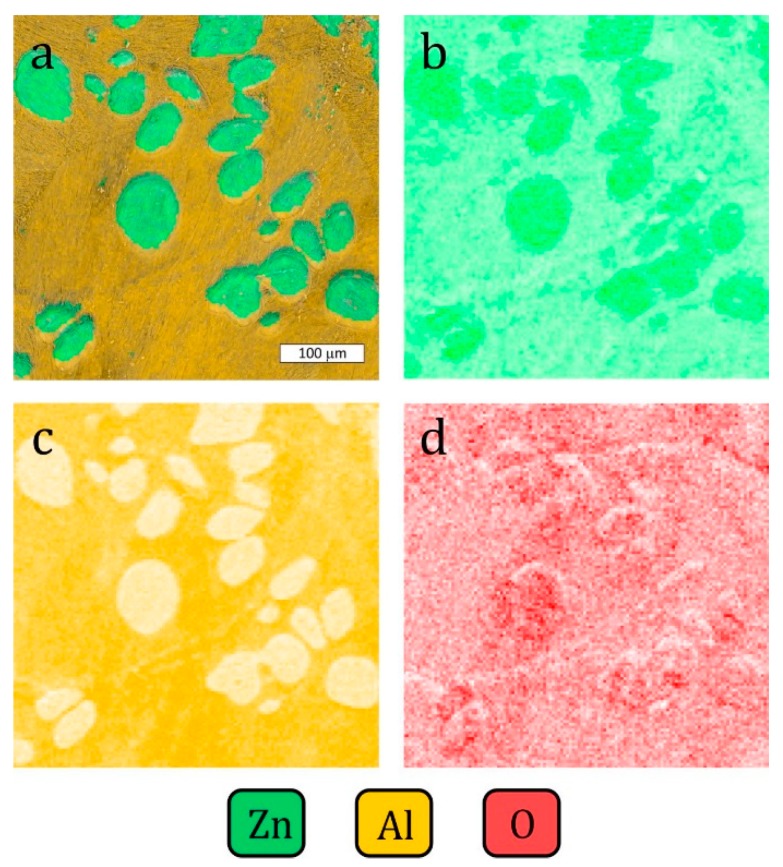
SEM and EDX images showing the distribution of elements on the sample surface after polarization up to the potential +300 mV vs. E_corr_: (**a**) SEM image merged with zinc and aluminum, (**b**) zinc, (**c**) aluminum, (**d**) oxygen. Element-rich areas are darker in the image.

The sample polarized up to a potential of +300 mV vs. E_corr_ experienced more extensive corrosion of the eutectic areas over its entire surface (Figure 10). At this stage, the dissolution of the η phase and the revealed α crystals (or products of its corrosion), was observed at the macroscopic scale (Figure 11). This is consistent with the observations that the lamellar structure enables the storage of corrosion products in areas of the corroded α phase, thereby delaying the corrosion process in the eutectic η phase [36]. A higher oxygen content was observed in the dendritic regions (Figure 12). The dendritic η phase dissolved and underwent anodic dissolution reactions [29] which resulted in a constant increase of the current density with the increased polarization potential (Figure 6).

**Figure 13:**
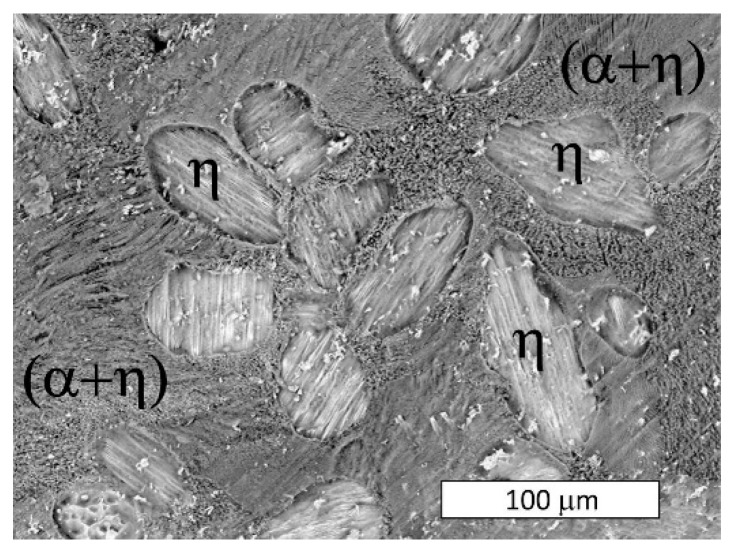
The surface of a sample after polarization up to a potential of +450 mV vs. E_corr_. Changes on the sample surface due to corrosion are visible over the entire alloy surface. SEM.

**Figure 14:**
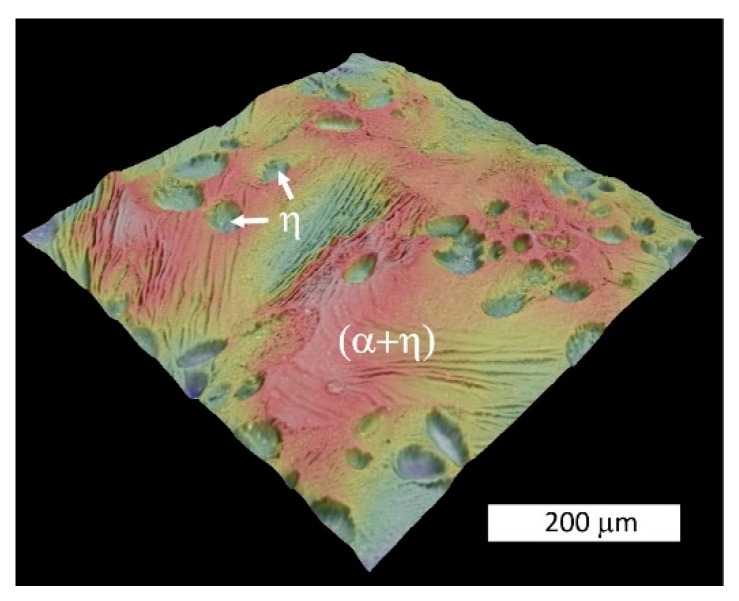
3D SEM topography of the sample surface after polarization up to a potential of +450 mV vs. E_corr_. The image based on “shape from shading” technology shows the selective dissolution of the η-phase and a macroscopic exposure of the eutectic structure. Blue indicates the lowest areas, while red represents the highest features.

After reaching a potential of −675 mV vs. Ag/AgCl (+450 mV vs. E_corr_) the current density decreased on the potentiodynamic curve (Figure 6). The SEM observations of samples tested at a higher potential revealed that at this stage, corrosion extended to all structural constituents (Figure 13). Due to the selective dissolution of the η phase and the macroscopic exposure of (α + η) eutectoid areas, surface topography was observed (Figure 14). These microscopic observations suggest the anodic character of the η phase relative to the α phase in the corrosive solution at this stage of corrosion. Zn^+^ ions are formed in the Zn-rich phase (η) due to anodic reactions: Zn → Zn^2+^ + 2e^−^, while the Al-rich phase (α) is expected to be responsible for the cathodic reactions: O_2_ + 2H_2_0 +4e^−^ → 4OH^−^. This indicates that despite the initiation of corrosion in areas of the α phase, there is a change in the η phase polarity and its corrosion. This is most likely due to the formation of a corrosion product film on the surface of the α phase that protects it from further corrosion, in accordance with other works [18,24,34]. Thus, changing the anodic zone polarity due to the formation of a protective film can be used in corrosion protection [38].

## 4. Discussion

The results were intended to discuss the effect of the cooling rate of a Zn-4Al alloy on the corrosion processes at the eutectoid transformation temperature. The main comments are as follows:

1) The microstructure of the material was typical for hypoeutectic cast Zn-Al alloys and was composed of a dendritic η-phase: Zn(Al) solid solution and lamellar (α + η) eutectoid. It contained the products of the eutectoid reaction which transformed the γ phase to (α + η) at 275 °C. Increasing the susceptibility to corrosion by increasing the aluminum content in Zn-Al alloys has been reported in the previous literature [5,19] which may be associated with an increased volumetric fraction of the (α + η) eutectic. Upon progression of the corrosion process, the (α + η) eutectoid structure in eutectic areas was attacked first and subjected to intense corrosion. Therefore, increasing the eutectic volumetric fraction should deteriorate the corrosion resistance of Zn-Al alloys. The high corrosion tendency of eutectic areas may induce intergranular corrosion [10,22,23]. In the case of two cooperating details, the accompanying pulverisation promotes the penetration of material fragments and the corrosion products into the friction area [22,23].
2) Different cooling rates affected the hardness of samples annealed at 300 °C. Water quenching promoted the creation of a finer (α + η) eutectoid structure from the γ phase in eutectic areas of the Zn-Al alloy and obtained higher hardness values. Slower cooling formed a coarser eutectic structure in the alloy, which translated into a lower hardness. After furnace cooling, a hardness similar to the as-cast material was obtained. Heat treatment at 250 °C showed no effect on the hardness of the Zn-4Al alloy.
3) A finer eutectoid structure decreased the corrosion current density I_corr_ compared with a coarse structure, which indicates that the short phase distances of eutectoid structures may contribute to the protection of the anode phase and reduce the corrosion rate. The corrosion potential E_corr_ remained rather constant, although a slight decrease was observed.
4) In the initial corrosion stage, the α-phase Al-base solid solution served as the anode in a formed corrosion microcell in the examined corrosive environment. As corrosion further developed, it extended over the entire alloy surface. Thus, it can be stated that the dissolution of the η phase was the preferred corrosion mode due to anodic dissolution reactions. This phenomenon may have been related to the formation of an α-phase corrosion product film. The formation of this film can also explain the lower corrosion current density due to a decrease in the cathode activity due to a smaller distance between eutectoid components.
5) If there is an anode phase whose fragments are fine and homogeneously distributed within the grain, corrosion will lead to their dissolution and the material eventually becomes quasi-homogeneous. A very different situation takes place for large η phase dendrites which occurs in the microstructure of Zn-Al alloy. In this case, corrosion develops involving these structural elements, which decrease the cross-sections of components made of this material.

## 5. Conclusions

A finer eutectoid structure was shown to improve the corrosion resistance of the Zn-4Al alloy, which indicates that the small phase distances between the eutectoid structures may help protect the anode phase. Importantly, this was also accompanied by an increase in the alloy hardness. This issue is important from the point of view of the heat treatment design of machine components exposed to chloride ions.

**Figure 15:**
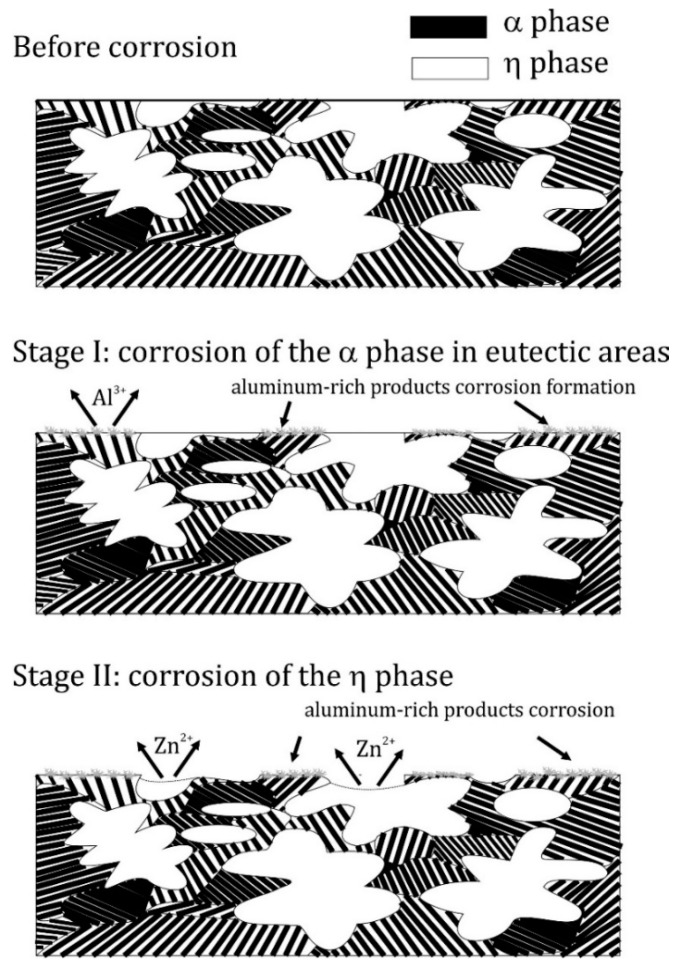
Schematic illustration of the corrosion mechanism based on as-delivered Zn-4Al alloy.

Although corrosion was initiated in the α-phase, the polarity of the η-phase changed, and its corrosion was observed. This was most likely due to the formation of a corrosion product film on the surface of the α phase that protected it from further corrosion. A scheme of the corrosion mechanism is presented in Figure 15. 

## Figures and Tables

**Table 1:** Scheme of the research design.

Material		Methods
As delivered		Hardness measurements, Microstructural examination, Electrochemical examination, SEM surface evaluation
Heat treatment at 250 °C	Furnace cooled	Hardness measurements
	Air cooled	
	Water quenched	
Heat treatment at 300 °C	Furnace cooled	Hardness measurements, Microstructural examination, Electrochemical examination, SEM surface evaluation
	Air cooled	
	Water quenched	

**Table 2:** The electrochemical parameters obtained for as delivered samples, as well as samples heat-treated at 300 °C and subjected to different cooling rates.

Sample	I_corr_ (µA/cm^2^)	E_corr_ (V) vs. Ag/AgCl	E_OCP_ (V)
As delivered	9.45 ± 0.36	−1.05 ± 0.01	−1.02 ± 0.01
Furnace-cooled	7.01 ± 0.23	−1.06 ± 0.01	−1.02 ± 0.01
Air-cooled	5.47 ± 0.9	−1.06 ± 0.01	−1.03 ± 0.01
Water-quenched	4.74 ± 0.20	−1.07 ± 0.01	−1.05 ± 0.02

**Table 3:** Chemical composition obtained from EDX analysis of point 1 in Figure 7a.

Element	Atomic %	Weight %
Zn	22.15	45.88
Al	39.14	33.47
O	37.04	18.78
Cl	1.67	1.87
